# Wearable Biosensors in Youth With Autism With High Behavioral Acuity: Supportive Practices, Acceptability, and the Importance of Nonverbal Signs of Assent to Research Participation

**DOI:** 10.1080/19447515.2025.2583714

**Published:** 2025-12-19

**Authors:** Briana J. Taylor, Christine B. Peura, Erin R. Riddell, William R. Strathmann, Jessie B. Northrup, Carla A. Mazefsky, Yasmin Andalib, Matthew Siegel

**Affiliations:** a The Roux Institute at Northeastern University, Portland, ME;; b Northeastern University, Boston, MA;; c MaineHealth Institute for Research, Scarborough, ME;; d Maine Behavioral Healthcare, Spring Harbor Hospital, Westbrook, ME;; e Greater Portland Health, Portland, ME;; f University of Pittsburgh Medical Center, Pittsburgh, PA;; g Tufts University School of Medicine, Boston, MA;; h Boston Children’s Hospital, Boston, MA;; i Harvard Medical School, Boston, MA

**Keywords:** Autism, wearables, biosensors, physiology, verbal ability

## Abstract

Wearable biosensors can provide insight into the internal states of individuals with autism with communication challenges; however, sensory sensitivities may make wearable biosensors uncomfortable. We describe our approach for introducing a wrist-worn biosensor (Empatica E4) to participants and using supportive techniques. Most participants (76.6%) were able to wear the biosensor for a duration of 15 minutes. The option to wear a colorful sweatband over the biosensor, verbal encouragement, and edible rewards were found to be helpful. Data collection was unsuccessful for the 23.4% of participants who did not accept wearing the device, which was more common for minimally speaking participants. Tailored approaches and attention to nonverbal signs of assent or discomfort are essential for conducting wearable biosensor research with individuals with autism.

## Résumés en Français

Les biocapteurs portables peuvent révéler desinformations sur l’état interne des personnes autistes ayant desdifficultés de communication; cependant, leurs sensibilitéssensorielles peuvent rendre les biocapteurs portables inconfortables.Nous décrivons notre approche pour introduire un biocapteur portéau poignet (Empatica E4) auprès des participants et pour utiliserdes stratégies de soutien. La plupart des participants (76,6 %) ontréussi à porter le biocapteur pendant 15 minutes. L’option deporter un bandeau coloré par-dessus le biocapteur, lesencouragements verbaux et les récompenses comestibles se sont avérésutiles. La collecte de données a été un échec pour les 23,4 % desparticipants qui ont refusé de porter le dispositif, ce qui étaitplus fréquent chez les participants minimalement verbaux. Desapproches personnalisées et une attention particulière aux signesnon verbaux d’assentiment ou d’inconfort sont essentielles pour menerdes recherches sur les biocapteurs portables auprès de personnesautistes.

## Resúmenes al Español

Los biosensoresportátiles pueden ofrecer información sobre los estados internos depersonas autistas con dificultades de comunicación; sin embargo, lassensibilidades sensoriales pueden hacer que estos dispositivosresulten incómodos. Se describió el enfoque para introducir unbiosensor de muñeca (Empatica E4) a los participantes y las técnicasde apoyo utilizadas. La mayoría de los participantes (76,6%) pudousar el biosensor durante un periodo de 15 minutos. Se encontró quela opción de cubrir el biosensor con una muñequera colorida,brindar estímulos verbales y usar recompensas comestibles fueronestrategias útiles. La recolección de datos no fue exitosa en el23,4% de los participantes que no aceptaron usar el dispositivo, locual fue más común en aquellos con comunicación verbal mínima.Los enfoques personalizados y la atención a las señales no verbalesde asentimiento o incomodidad son esenciales para realizarinvestigaciones con biosensores portátiles en personas autistas.

## Background

Youth with autism spectrum disorder (ASD) often face challenges in social communication, making it difficult to report internal states like pain or emotional distress. This is particularly problematic for those with profound autism who are minimally speaking or those with limited nonverbal expressivity during affective states. In the absence of reliable verbal or nonverbal cues, caregivers are frequently left to interpret internal states, contributing to increased risk for unpredictable aggressive or self-injurious episodes and reduced ability to intervene proactively (Bronsard et al., 2010). Objectively measured biomarkers of internal states may help bridge communication gaps and enable preventative interventions.

Wearable biosensors offer an opportunity to objectively monitor changes in autonomic physiology (e.g., heart rate variability). While autonomic physiology is affected by various activities (e.g., exercise, sleep) it can also vary with emotional states (Hachenberger et al., 2023). For instance, sympathetic arousal is associated with increased psychosocial stress while parasympathetic activation is associated with increased emotion regulation (Kim et al., 2018). As such, physiological signals may serve as useful correlates of internal states, especially when interpreted within the context of behavioral events. Increasingly, ASD researchers are leveraging computational methods to predict the onset of challenging behaviors from physiological signal data (Imbiriba et al., 2023; Zwilling et al., 2022). If algorithms applied to physiological data can accurately predict future challenging behaviors, they may be integrated into mobile systems to alert caregivers and other support professionals of imminent behaviors.

Wearable biosensors hold promise for improving behavioral health monitoring in ASD, but their use must be guided by ethical and inclusive research practices. Individuals with profound autism or high behavioral acuity are often underrepresented in research due to logistical and sensory challenges (Stedman et al., 2019). Sensory sensitivities, common in up to 90% of those with ASD (Marco et al., 2011), may limit biosensor tolerability. While systematic desensitization is appropriate for medical procedures (Martínez Pérez et al., 2023), less intensive, participant-centered approaches are preferable for research (Cano et al., 2024). This report shares our experiences implementing a wearable biosensor protocol with youth with autism requiring high levels of support to inform future inclusive research practices.

## Methods

Data were collected as part of the Autism Inpatient Collection (AIC), a multisite study focused on youth with autism and developmental disorders receiving inpatient psychiatric care for challenging behavior (Siegel et al., 2015). Data presented here were collected at three AIC sites as part of a substudy to evaluate physiological precursors of challenging behavior. The study protocol was approved by each data collection site’s Institutional Review Board and written informed consent was provided by each participant’s legal guardian.

Participants were 77 youth (5 to 21 years old), with a clinical ASD diagnosis and empirically derived cutoff scores on the Autism Diagnostic Observation Schedule, Second Edition (ADOS-2) (Lord et al., 2012). Eligible participants had caregiver-reported aggression or self-injury as the reason for hospitalization, as the purpose of the broader study was to evaluate the utility of physiological signals to predict aggressive and self-injurious behaviors. The E4 from Empatica was used to collect physiological signal data, including heart rate, heart rate variability, and electrodermal activity.

We developed a flexible approach for acclimating participants to the wearable biosensor. Our approach drew from sensory integration practices, speech language pathology, and positive behavioral reinforcement to support engagement and reduce avoidance (Schmidt et al., 2013; Schoen et al., 2019). The protocol was developed collaboratively through a series of meetings between AIC site principal investigators, nonresearch unit specialists (e.g., occupational therapists, speech-language pathologists, behavior analysts, and nursing staff), and research assistants responsible for data collection. We used an iterative, feedback-driven process to refine our approach based on observations and testimonies of research assistants applying the approach in practice.

### Device introduction procedure:

Devices were introduced to participants at times when they tended to present fewer challenging behaviors during their hospital stay, per clinical team observations. Research staff introduced themselves and explained the study in simple terms using the participant’s preferred communication method (e.g., devices, gestures, or pictures). Participants were given time to explore the biosensor. Research staff asked to place the biosensor on participants’ wrists while monitoring verbal and nonverbal signs of discomfort. Verbal signs included objections (e.g., “No,” “I don’t want to”) or increased vocalizations with negative valence. Nonverbal signs included actions directed at the biosensor (e.g., attempting to take it off, scratching at it) and physical avoidance of the research team members (e.g., turning away or hiding when the team approached). If signs of discomfort were noted, supportive strategies were used but discontinued if discomfort persisted.

### Supportive techniques included:

1. **Verbal encouragement**: Preferred staff provided verbal encouragement.
2. **Wearable accessories**: Participants were given the choice to cover the biosensor with colorful sweatbands or sleeves, choosing the type, color, and number to enhance their comfort and agency.
3. **Behavioral reinforcement**: Earned rewards (e.g., edibles, iPad time) were used when appropriate.
4. **Visual supports**: Social stories, schedules, or “First/Then” boards were utilized to illustrate the steps involved in wearing the biosensor and provided clarity.

If participants wore the biosensor without signs of discomfort or withdrawal of assent, research staff logged the start time of data collection and monitored participants’ comfort throughout. Staff tracked reasons for ending sessions and aimed to extend gradually data collection times. Participant safety was prioritized across all phases. If distress or unsafe behaviors related to the device arose, the protocol was discontinued. The biosensor device was considered acceptable if participants completed at least one session with 15 minutes of data collected without issue.

#### Measures and Assessments

At admission, caregivers provided demographic information and completed questionnaires to measure a participant’s behavioral challenges. Study staff conducted diagnostic and nonverbal intellectual quotient (IQ) assessments.

#### Statistical Analysis

Descriptive statistics were used to characterize the sample. Analysis of variance and χ^2^ tests examined differences between those who did and did not find the wearable biosensor acceptable across continuous and dichotomous variables, respectively. A statistical significance threshold of α = 0.01 was used to control for multiple comparisons and minimize Type I error.

## Results

The 77 participants (mean age = 12.0 ± 3.5) were mostly male (89.6%), White (88.3%), and non-Hispanic (90.4%) (Table 1). The mean nonverbal IQ was 71.4 ± 25.3, 51.9% of the sample was minimally speaking, and mean ADOS-2 comparison scores were 8.1 ± 1.4. Of the 77 participants, 59 (76.6%) were able to ultimately wear the biosensor. Eighteen participants (23.4%) rejected the biosensor, removed it, or showed distress after it was applied. Minimally speaking youth were significantly less likely to accept the biosensor compared to fluently speaking youth (χ^2^ = 4.3, *p* = .04). However, of the 40 minimally speaking participants, approximately 60% were able to wear the biosensor. There were no other demographic or clinical differences between those who did and did not wear the biosensor. Of the 59 participants who wore the biosensor, 79.4% chose to cover the biosensor with a sweatband. Encouragement from familiar staff members and edible rewards were helpful for 29.4% and 17.6% of participants, respectively.

## Discussion

Most participants in our sample tolerated the wearable biosensor with the help of low-burden, individualized supports. However, a subset who were more likely to be minimally speaking did not, highlighting the need to attend closely to nonverbal signs of assent and the sensory burden of wearable devices. Given the frequent exclusion of this group from research (Stedman et al., 2019), developing acceptable procedures is essential. While wrist-worn devices are generally well tolerated (Kim et al., 2018), refinements are needed to improve accessibility across the autism spectrum. Evaluating alternative formats, such as smart textiles or minimally perceptible devices, is also needed.

Most participants chose to cover the biosensor with a sweatband, which may have increased acceptability by increasing the agency of participants or reducing awareness of the device. This aligns with prior research showing that cosmetic customization can improve comfort with wearable devices (Kang et al., 2017). In populations with autism, both users and caregivers emphasize the importance of unobtrusive, personalized designs (Xifaras et al., 2025). Personalized motivators (e.g., earned iPad time) also increased acceptability, highlighting the value of individualized approaches for initial acceptance of biosensors. Longitudinal studies are essential to understand how wearable biosensors could be used for day-to-day clinical applications, as limited existing research on long-term adherence exists (Garcia et al., 2021).

Wearable biosensors offer valuable insight into internal states in youth with autism but must be introduced in ways that respect sensory sensitivities and communication differences. Voluntary participation requires attention to behavioral signs of assent and dissent. Low-burden, individualized strategies can improve acceptability and support the inclusion of underrepresented populations in autism research.

## Figures and Tables

**Table 1.** Sample characteristics

	Total sample (*N* = 77)	Tolerated biosensor (*n* = 59)	Did not tolerate biosensor (*n* = 18)	Sig.
**Demographic characteristics**				
Age, mean *(SD)*	12.0±3.5	11.9±3.5	12.1±3.9	0.06
Sex, male (%)	69(89.6)	51(86.4)	18(100.0)	2.72
Race, White (%)	68(88.3)	(89.8)	15(83.3)	0.56
Ethnicity, non-Hispanic (%)	66(85.7)	52(88.1)	14(77.8)	0.20
**Clinical profile**				
Nonverbal IQ^a^, mean (*SD*)	71.4±25.3	74.0±24.8	60.8±25.5	2.94
Minimally speaking, *N* (%)	40(51.9)	24(40.7)	16(88.9)	12.84***
ADOS-2^b^ comparison score, mean (*SD*)	8.1±1.4	8.1±1.4	8.3±1.4	0.20
Length of hospital stay, mean days (*SD*)	38.4±33.6	37.2±35.9	43.4±23.2	0.41
**Psychiatric and behavioral impairment**				
Internalizing symptoms^c^, t score (*SD*)	65.9±9.1	66.8±8.6	63.5±10.2	1.37
Externalizing symptoms^c^, *t*score (*SD*)	68.8±7.3	69.6±7.8	66.4±5.1	1.99
ABC–Irritability subscale^d^, mean (*SD*)	28.9±8.6	27.9±9.4	32.1±4.2	2.77
Emotion dysregulation^e^–Reactivity, mean (*SD*)	58.4±7.8	59.1±8.6	56.2±4.0	1.52
Emotion dysregulation^e^–Dysphoria, mean (*SD*)	57.2±8.4	57.2±8.6	57.0±8.0	0.00

*Note*. Significance statistics were χ^2^s for categorical variables and *F*-statics for continuous variables;

*** *p* < .001.

a Leiter International Performance Scale, 3rd Edition (Roid et al., 2013);

b Autism Diagnostic Observation Schedule, 2nd Edition (Lord et al., 2012);

c Child Behavioral Checklist (Achenbach & Edelbrock, 1991);

d Aberrant Behavior Checklist (Aman et al., 1985);

e Emotion Dysregulation Inventory (Mazefsky, 2021).
